# Moyamoya disease-associated protein mysterin/RNF213 is a novel AAA+ ATPase, which dynamically changes its oligomeric state

**DOI:** 10.1038/srep04442

**Published:** 2014-03-24

**Authors:** Daisuke Morito, Kouki Nishikawa, Jun Hoseki, Akira Kitamura, Yuri Kotani, Kazumi Kiso, Masataka Kinjo, Yoshinori Fujiyoshi, Kazuhiro Nagata

**Affiliations:** 1 Laboratory of Molecular and Cellular Biology, Faculty of Life Sciences, Kyoto Sangyo University, Kyoto 603-8555, Japan; 2 Cellular and Structural Physiology Institute, Nagoya University, Nagoya, 464-8601, Japan; 3 Research Unit for Physiological Chemistry, The Center for the Promotion of Interdisciplinary Education and Research, Kyoto University, Kyoto, 606-8502, Japan; 4 Division of Applied Life Sciences, Graduate School of Agriculture, Kyoto University, Kyoto, 606-8502, Japan; 5 Laboratory of Molecular Cell Dynamics, Faculty of Advanced Life Science, Hokkaido University, Sapporo, 001-0021, Japan

## Abstract

Moyamoya disease is an idiopathic human cerebrovascular disorder that is characterized by progressive stenosis and abnormal collateral vessels. We recently identified mysterin/RNF213 as its first susceptibility gene, which encodes a 591-kDa protein containing enzymatically active P-loop ATPase and ubiquitin ligase domains and is involved in proper vascular development in zebrafish. Here we demonstrate that mysterin further contains two tandem AAA+ ATPase modules and forms huge ring-shaped oligomeric complex. AAA+ ATPases are known to generally mediate various biophysical and mechanical processes with the characteristic ring-shaped structure. Fluorescence correlation spectroscopy and biochemical evaluation suggested that mysterin dynamically changes its oligomeric forms through ATP/ADP binding and hydrolysis cycles. Thus, the moyamoya disease-associated gene product is a unique protein that functions as ubiquitin ligase and AAA+ ATPase, which possibly contributes to vascular development through mechanical processes in the cell.

Moyamoya disease is an idiopathic human cerebrovascular disorder in which intracranial internal carotid artery and its main branches are specifically and progressively occluded by the hyperplasia of vascular smooth-muscle cells and luminal thrombosis^1^,^2^,^3^. The arterial stenosis reduces blood flow and subsequently stimulates the compensatory development of characteristic collateral small vessels, which appear as a “puff of smoke” on the cerebral angiography^2^,^3^,^4^. On the basis of the characteristic appearance, the disease was termed “moyamoya”, which is an onomatopoeia meaning “puff of smoke” in Japanese^5^. The two major symptoms of moyamoya disease are brain ischemia and intracranial hemorrhage^2^,^3^. Although the etiology remains largely unclear, epidemiologic studies have suggested that ethnic and genetic factors contribute to the disease onset. The prevalence and incidence of moyamoya disease are the highest in East Asia, particularly in Japan^2^. The incidence in Japan is approximately 10-fold higher than that in Europe^6^. 3.16–10.5 of 100,000 Japanese individuals contracted the disease^7^,^8^,^9^. 10%–15% of Japanese patients have a family history of the disease^10^. Familial moyamoya disease is inherited in an autosomal dominant manner with incomplete penetrance in Japan^11^.

Several potential susceptibility loci for familial moyamoya disease had been suggested by genetic studies^12^,^13^. Our group and others recently succeeded for the first time in identifying a specific susceptibility gene for moyamoya disease on chromosome 17q25.3 through genome-wide linkage analyses^14^,^15^. The gene encodes a novel huge 591-kDa protein. Rare missense variants in its coding region significantly increase the risk of the disease and are associated with vascular abnormalities^14^,^15^,^16^,^17^,^18^,^19^. Its cloning and characterization revealed that the huge protein contains enzymatically active Walker A and B motifs and a RING (really interesting new gene) finger domain^14^. Walker A and B motifs are nucleotide- and magnesium-binding motifs, respectively, and cooperatively function as the most ubiquitous NTPase domain, which is also known as P-loop NTPase domain^20^. The RING finger domain is a well-established ubiquitin ligase domain that covalently modifies substrate proteins with ubiquitin and stimulates intracellular biological processes such as proteasomal protein degradation^21^,^22^. In the current study, we further demonstrated that this protein contains a pair of AAA+ (ATPases associated with diverse cellular activities) ATPase modules. AAA+ ATPases are defined by specific structures surrounding Walker A and B motifs and thus are a subclass of Walker-type NTPases. Historically, proteins that contain conserved amino acid sequence surrounding the Walker motifs were first defined as AAA ATPases, and a group of proteins forming secondary and tertiary structures similar to the AAA module was subsequently defined as AAA+ ATPases^23^,^24^. AAA+ ATPases, including classic AAA ATPases, exhibit characteristic activities that lead to formation of a ring-shaped hexameric oligomer and change their overall structure during ATP binding and hydrolysis cycles^23^,^24^. Representative examples of this class of ATPases are the base ATPase subunits of proteasome, ClpB/Hsp104, p97, NSF (N-ethylmaleimide sensitive fusion protein), katanin, SV40 large T antigen, and dynein^23^,^24^. They mediate various mechanical processes in the cell, such as protein unfolding, polypeptide threading through the central pore of the ring-shaped oligomer, membrane fusion, disassembly of protein complexes, DNA unwinding, cargo transport, and flagellar movement. Thus, one could argue that AAA+ ATPases are a sort of ATP-burning molecular engine. Although the newly identified susceptibility gene for moyamoya disease and its encoded product had been automatically registered as RING finger protein 213 (RNF213) by databases, we here propose a more realistic name: moyamoya steno-occlusive disease-associated AAA+ and RING finger protein (mysterin).

Mysterin is an intracellular soluble protein that is located in the cytosol and partially associates with unidentified intracellular structure^14^. The mysterin gene is likely conserved across vertebrates and is ubiquitously expressed in human and mouse tissues^14^,^15^. Although mysterin exhibits enzymatic activities such as ATPase and ubiquitin ligase^14^, its intracellular function and mode of action remain largely unclear. The fact that mysterin was identified in a human familial cerebrovascular disease suggests its contribution to vascular structure and/or function. In fact, knockdown of zebrafish mysterin using morpholino antisense oligonucleotide specifically and significantly impairs the vascular network and leads to excess angiogenesis, clearly indicating that mysterin plays a crucial role in proper vascular development^14^. Although the biological effects of the disease-associated missense variants have not yet been clarified, they possibly interfere with the intracellular function of mysterin and increase the risk of moyamoya disease.

In this report, we first demonstrate that mysterin contains a pair of AAA+ ATPase modules and forms a huge ring-shaped oligomeric complex, the size of which is estimated to be comparable with that of known supramolecular complexes such as the ribosome. This complex dynamically changes its oligomeric state through ATP-binding and hydrolysis cycles. Hence, the moyamoya disease-associated gene product mysterin is a unique protein that exhibits ubiquitin ligase and AAA+ ATPase activities, which possibly mediate biophysical processes in the cell.

## Results

### Mysterin contains tandem AAA+ ATPase modules

**Figure 1:**
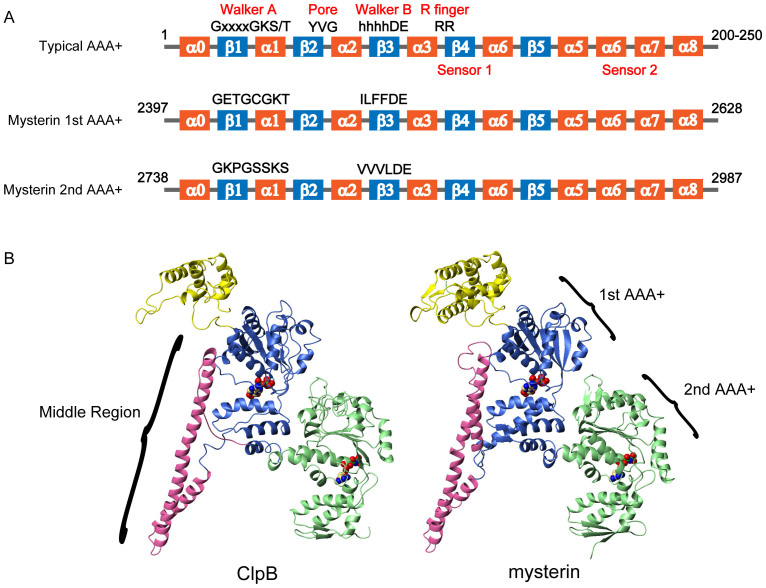
Potential two AAA+ modules of mysterin. (A) Predicted secondary structures surrounding Walker A and B motifs of mysterin resemble those of typical AAA+ ATPases, which contains indicated secondary structures (α and β refer to α helix and β strand, respectively) and elements: Walker A (x indicates any amino acid) and B (h indicates hydrophobic residue) motifs, pore region, arginine finger, and sensor 1 and 2 regions. Definitions of the structural elements are based on Ref. 24. (B) Homology model of AAA+ modules of mouse mysterin based on a crystal structure of *Thermus thermophilus* ClpB (PDB ID: 1QVR).

We previously identified a set of Walker A and B NTPase motifs in mysterin and demonstrated their combined ATPase activity^14^. Thereafter, we made a prediction of the secondary structure of the entire 5207 amino acid sequence of mysterin and found a potential characteristic organization of secondary structures surrounding the Walker motifs (Supplementary Fig. 1A and B). The deduced secondary structure significantly resembles that of AAA+ ATPase (Fig. 1A and Supplementary Fig. 1A). Furthermore, we found another potential AAA+ module containing another set of Walker A and B motifs following the first AAA+ module (Fig. 1A and Supplementary Fig. 1B). Such a tandem organization of AAA+ modules is characteristic of several known AAA+ ATPases, including ClpA-D, Hsp104, p97, and NSF. Fig. 1B shows a homology model of mysterin's tandem AAA+ modules based on a crystal structure of bacterial ClpB (generated by RIKEN FAMSBASE: http://fams.bio.chuo-u.ac.jp/RIKEN/). Intriguingly, the model suggests that a long (approximately 110 amino acids) and helical middle region between the first and second AAA+ modules of mysterin resembles that of bacterial ClpB and its yeast homologue Hsp104. ClpB and Hsp104 are molecular chaperones that physically dissolve protein aggregates and contribute to cellular proteostasis through mechanical activity of the AAA+ modules^25^,^26^. The middle region of ClpB and Hsp104 is not merely a spacer connecting the two AAA+ modules, but regulates their disaggregation activities^26^,^27^,^28^,^29^. The characteristic long and helical middle region of mysterin could coordinate the two AAA+ ATPase modules.

### Two AAA+ ATPase modules exhibit enzymatic activities

**Figure 2:**
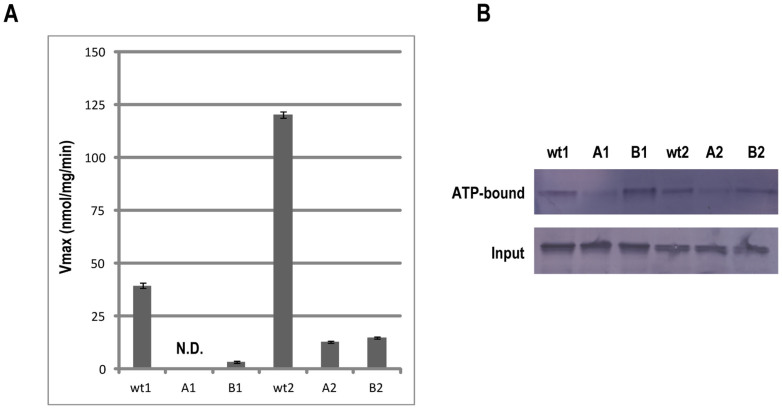
ATPase and ATP-binding activities of the first and the second AAA+ ATPase modules. (A) Affinity-purified N-terminally GST-fused first and second AAA+ modules were incubated with 5 mM ATP. The free phosphate released by ATP hydrolysis was measured by malachite green assay. Error bars represent the standard errors from the three independent experiments. (B) Affinity-purified N-terminally GST-fused first and second AAA+ modules were incubated with ATP agarose beads, and the bound protein was detected by Western blotting using anti-GST antibody. Full-length blots are presented in Supplementary Fig. 4A.

Prediction of the secondary structure of mysterin suggested an existence of unexpected two AAA+ ATPase modules. To examine if these potential ATPase modules are enzymatically active or not, we generated recombinant first and second AAA+ modules each of which is fused with glutathione S-transferase (GST) tag. The recombinant proteins were expressed in bacteria and purified using glutathione affinity agarose beads. The purified proteins were then incubated with ATP of saturating concentration (5 mM), and their ATP hydrolysis activities were measured by malachite green assay. Compared to the values obtained from the phosphate standard, ATPase activities of the first and the second AAA+ ATPase modules were evaluated at 39 and 120 nmol/mg/min, respectively (Fig. 2A).

A single AAA+ motif contains a set of Walker A and B motifs, which are essential for ATP binding and for hydrolysis of ATP, respectively. We introduced mutations into the conserved amino acids of the Walker motifs and tried to confirm the expected contribution of the Walker motifs to entire ATPase activity of the AAA+ modules. As expected, ATPase activities of the Walker A and B motif mutants (A1, B1, A2 and B2) were drastically decreased compared with those of the wild type proteins (Fig. 2A). Thus, intrinsic Walker motifs are essential for the ATPase activities of the two AAA+ ATPase modules of mysterin. Next, we examined ATP binding activities of the AAA+ modules. We incubated the purified AAA+ fragments with ATP agarose beads and evaluated binding of the proteins to the agarose beads. The first and the second AAA+ ATPase modules were bound to ATP agarose beads (Fig. 2B). Mutation in the Walker A motifs markedly decreased the binding of ATP, whereas the mutation in Walker B motifs did not affect ATP binding, indicating that the intrinsic Walker A motifs are essential for ATP binding activity of the AAA+ modules as reported for other AAA+ ATPases.

### Mysterin forms a huge ring-shaped oligomer

**Figure 3:**
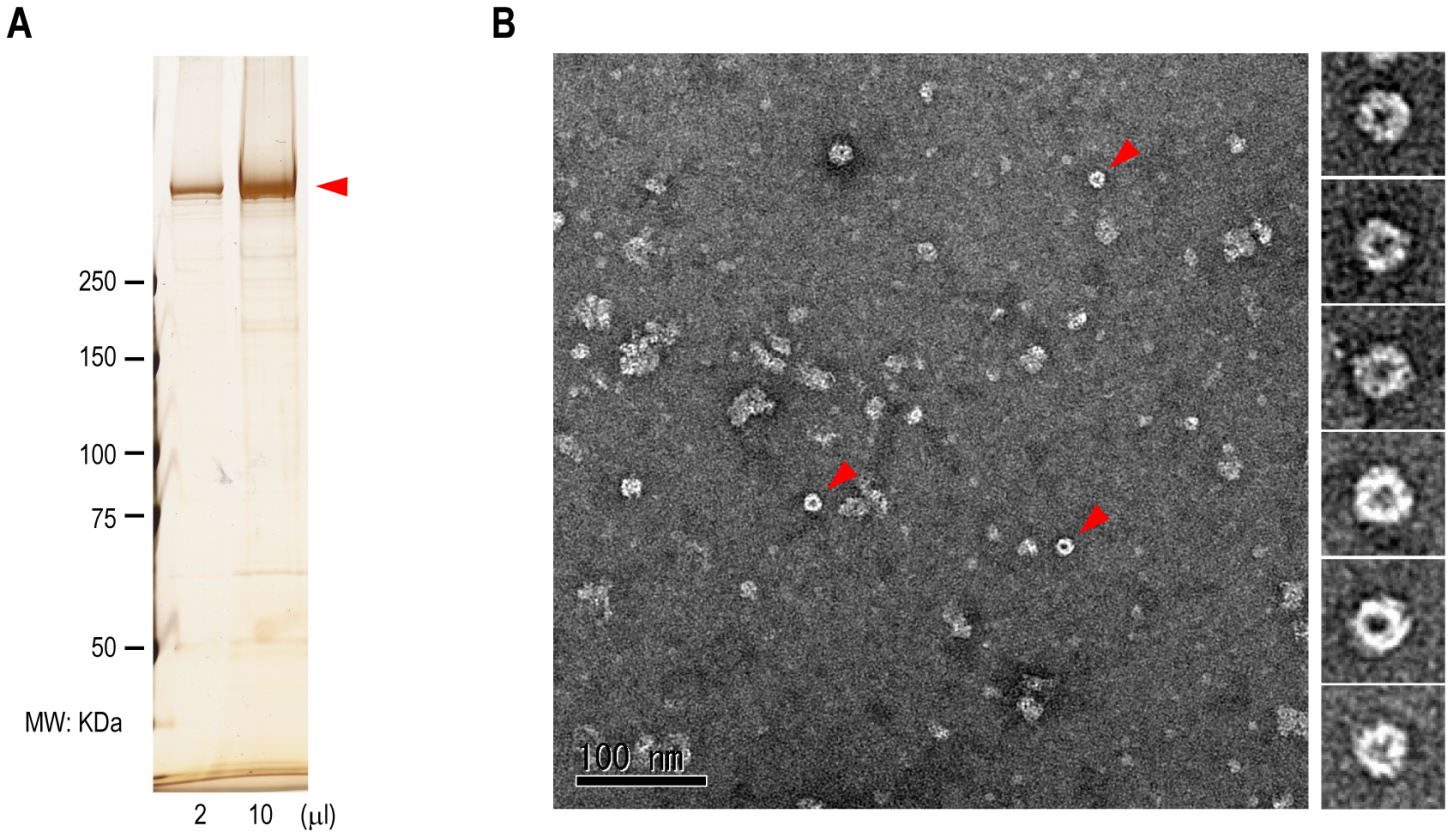
Electron micrographs of negative stained samples demonstrate ring-shaped oligomer of purified mysterin. (A) C-terminally 3 × FLAG-tagged mysterin was purified using anti-FLAG affinity agarose beads. The indicated volume of purified protein sample was applied to a 3%–10% gradient SDS gel and was silver stained. The arrowhead indicates the 591-kDa mysterin. (B) Electron micrographs of negative stained samples demonstrate ring-shaped particles.

The single most characteristic feature defining AAA+ ATPase is the ability to form a ring-shaped homo-oligomer, which is typically a homo-hexamer^23^,^24^. To directly address this issue, we transfected full-length mysterin the C-terminus of which was tagged with three tandem FLAG (3 × FLAG) epitope to human embryonic kidney (HEK)-293 cells. C-terminally-tagged mysterin was purified from the cells (Fig. 3A) and characterized using negative staining electron microscopy (EM). The result depicted particles of ring-shaped structures within μm square in a high density area (Fig. 3B), indicating that mysterin has the ability to form a ring-shaped oligomer in the cell. Mysterin is a huge protein with a molecular mass of 591 kDa, and thus, its potential hexamer should be approximately 3.5 MDa, which is comparable with that of known huge macromolecular complexes such as the ribosome. The diameter of the currently observed ring-shaped particles was between 17 and 20 nm, which could be comparable with the ~20-nm diameter of the prokaryotic ribosome^30^. However, the majority of the purified mysterin appeared to form denatured aggregates (Fig. 3B). These denatured forms could be attributed to their deformation in the processes of protein purification and/or negative staining for EM observation.

### Potential dynamic equilibrium of mysterin in the cell

Most AAA+ ATPases predominantly form homo-hexamers at the physiological concentration of ATP. However, the current EM observation demonstrated that a relatively small amount of mysterin molecules form homo-oligomers. For elucidating the oligomeric states of mysterin in vivo, we generated N-terminally green fluorescent protein (GFP)-tagged mysterin (GFP-mysterin) and attempted to investigate the states in the cell using FCS. This technique has been applied to assess biomolecular diffusion, homo-oligomerization, and interactions with other molecules in living cells with single-molecule sensitivity^31^. Confocal fluorescence microscopy confirmed that GFP-mysterin is localized to the cytosol in a mouse neuroblastoma Neuro2A cell (Supplementary Fig. 2A). The fluorescence of GFP-mysterin fluctuated, and no notable photobleaching was observed, indicating that GFP-mysterin is mobile in the cell (Supplementary Fig. 2B and C). The autocorrelation function of GFP-mysterin that was obtained using FCS was dramatically shifted to the right compared with that of control GFP (Supplementary Fig. 2D). This indicates that the mobility of mysterin-GFP is lower than that of intact GFP. To quantify the mobility of GFP-mysterin and intact GFP, we performed a two-component curve fitting analysis on the autocorrelation functions obtained. The curve fitting showed that most intact GFP (~94.9%) had a fast diffusion coefficient (36.4 μm^2^/s), corresponding with the previously reported value^32^ (Table 1), whereas approximately half of GFP-mysterin (43.6%) had a diffusion coefficient 13.4 μm^2^/s that was slower than that of intact GFP. From the Stokes–Einstein relation of these values, the molecular weight of mysterin was evaluated to be 548 kDa (see Materials and Methods). This value actually corresponds to that of monomeric GFP-mysterin (618 kDa). Thus, approximately half of GFP-mysterin diffuses as a monomer in the cell. The diffusion coefficient of the other fraction of GFP-mysterin was 0.49 μm^2^/s. The molecular weight of this fraction was evaluated to be 1.1 GDa, which was far larger than the estimated molecular weight of homo-hexamer of GFP-mysterin (~4 MDa). Such a huge in vivo complex possibly contains endogenous substrates and/or other cellular components. The potential homo-hexamer of GFP-mysterin is not able to be distinguished from such a fraction with a huge molecular mass. These in situ observations indicate that, unlike typical AAA+ ATPases, a relatively large amount of mysterin diffuses as a monomer in the cell and suggest that mysterin exists in an equilibrium condition between monomer and oligomer in the cell.

### The second AAA+ module contributes to disassembly of mysterin oligomer

**Figure 4:**
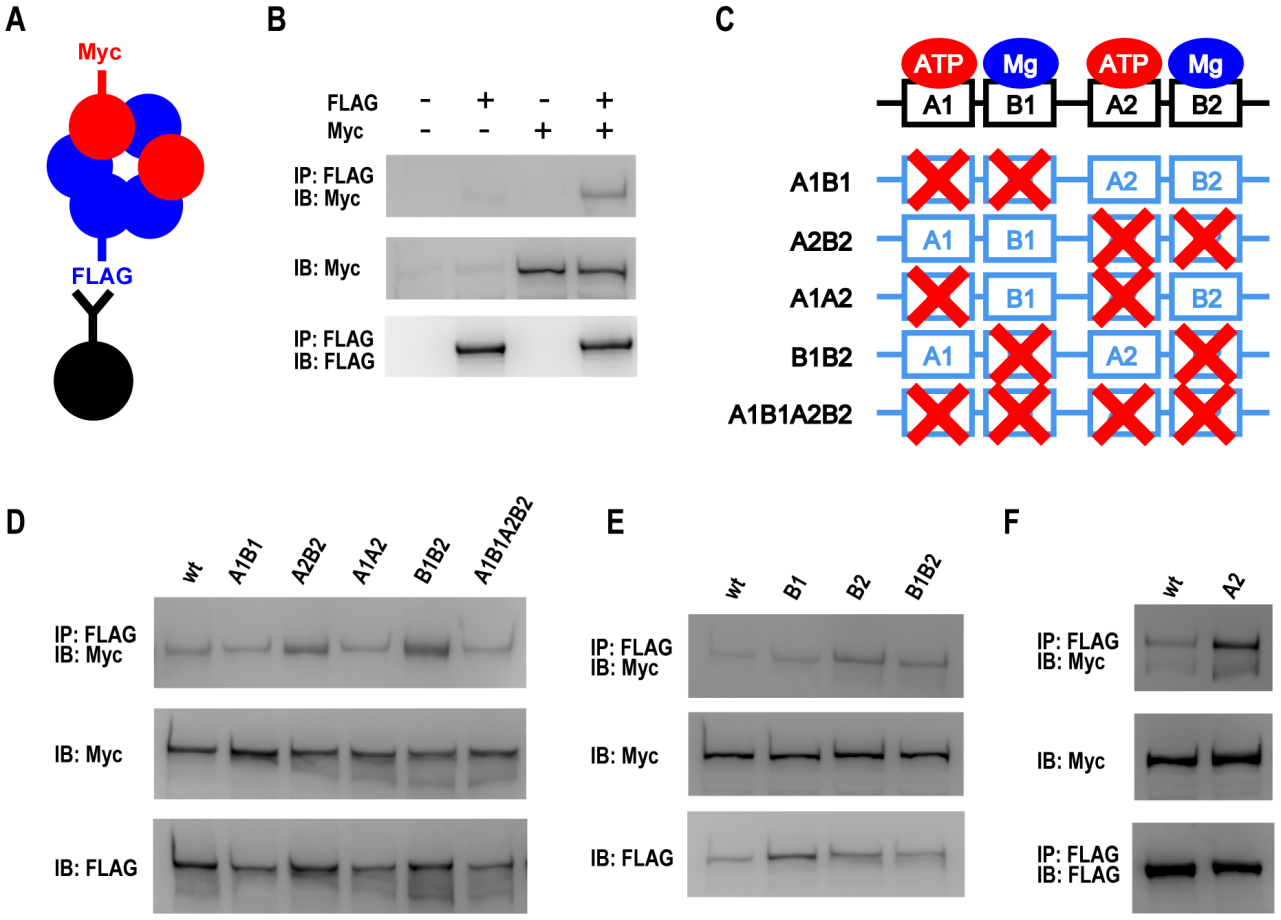
ATP hydrolysis of the second AAA+ module destabilizes mysterin homo-oligomers. (A) Schematic representation of the biochemical assay used for detecting mysterin homo-oligomer. (B) The complex of mysterin-3 × FLAG and mysterin-Myc was detected as schematically represented in (A). Full-length blots are presented in Supplementary Fig. 4B. (C) Schematic representation of AAA+ combination mutants of mysterin. A1, A2, B1, and B2 indicate the first and second Walker A motifs and the first and second Walker B motifs, respectively. (D) The A2B2 and B1B2 mutants exhibited significantly stronger homo-oligomer formation than did wild-type mysterin. Full-length blots are presented in Supplementary Fig. 4C. (E) The B2 single mutant solely showed enhanced homo-oligomer formation. Full-length blots are presented in Supplementary Fig. 4D. (F) The A2 single mutant solely showed enhanced homo-oligomer formation. Full-length blots are presented in Supplementary Fig. 4E.

To further clarify the ability of mysterin to form homo-oligomers, we designed a simple biochemical assay. When two differently tagged mysterin proteins are simultaneously expressed in cells, they would be stochastically involved in a same complex, which can be detected using immunoprecipitation (IP) and immunoblot analysis (Fig. 4A). C-terminally 3 × FLAG-tagged mysterin (mysterin-FLAG) and cMyc-tagged mysterin (mysterin-Myc) were simultaneously expressed in HEK-293 cells. Subsequently, the cell lysate was immunoprecipitated with anti-FLAG antibody, and the immunoprecipitate was immunoblotted with anti-Myc antibody (Fig. 4A). Co-IP of mysterin-FLAG and mysterin-Myc was detected as shown in Fig. 4B, clearly indicating that the two differently tagged mysterin proteins formed a single homo-oligomer. The efficiency of the co-IP was approximately 1%–3%, which was evaluated as the ratio of co-IP divided by the basal expression level. Such a low efficiency of co-IP, which reflected the efficiency of homo-oligomer formation, was consistent with the current EM observation as well as FCS analysis. In addition, we examined if the disease-associated amino acid change (R4810K) interfere with the oligomer formation using similar co-IP assay. As shown in Supplementary Fig. 3, R4810K mutation showed oligomer formation similar to that of wild type mysterin, suggesting that oligomer forming activity is not disturbed by disease-associated amino acid change.

ATP binding and hydrolysis cycles are generally correlated with overall structures and oligomeric states of AAA+ ATPases^33^,^34^,^35^,^36^. Therefore, we investigated the potential dynamic conversion of mysterin oligomer using various mysterin AAA+ mutants. As schematically shown in Fig. 4C, we generated mutant constructs that contained combined mutations at the conserved essential amino acids in the first Walker A and B motifs (A1B1), the second A and B motifs (A2B2), both A motifs (A1A2), both B motifs (B1B2), and all motifs (A1B1A2B2). Among them, the A2B2 and B1B2 mutants specifically exhibited enhanced co-IP (Fig. 4D), suggesting that crucial elements for oligomer dissociation are involved in those mutated motifs, and that mutation of those elements causes stabilization of the oligomer. The A2B2 and B1B2 mutants shared a mutation in the second B motif, which was thus estimated to be responsible for the oligomer stabilization. Therefore, we generated a B2 single mutant and examined the co-IP. As expected, the B2 mutant solely showed stronger co-IP (Fig. 4E). It is well established that a B mutation of an AAA+ module can lead to two effects. The primary effect is to eliminate the affinity of the B motif for magnesium ion. The secondary effect is the loss of ATPase activity, since magnesium binding to the B motif is necessary for ATPase activity of the entire AAA+ module. Thus, the stabilizing effect of the B2 mutation of mysterin could be due to either decreased magnesium binding or loss of ATPase activity. If the latter is the case, a similar effect should be observed when using a single mutant of the second A motif, because the A mutation primarily decreases nucleotide binding to the A motif, which further causes the loss of ATPase activity of the entire AAA+ module. As a result, the A2 single mutant of mysterin also showed enhanced co-IP (Fig. 4F), indicating that the loss of ATPase activity of the second AAA+ module was responsible for the oligomer stabilization. In other words, ATP hydrolysis in the second AAA+ module causes the dissociation of the mysterin ring-shaped oligomer.

### The first AAA+ module contributes to assembly of mysterin oligomer

**Figure 5:**
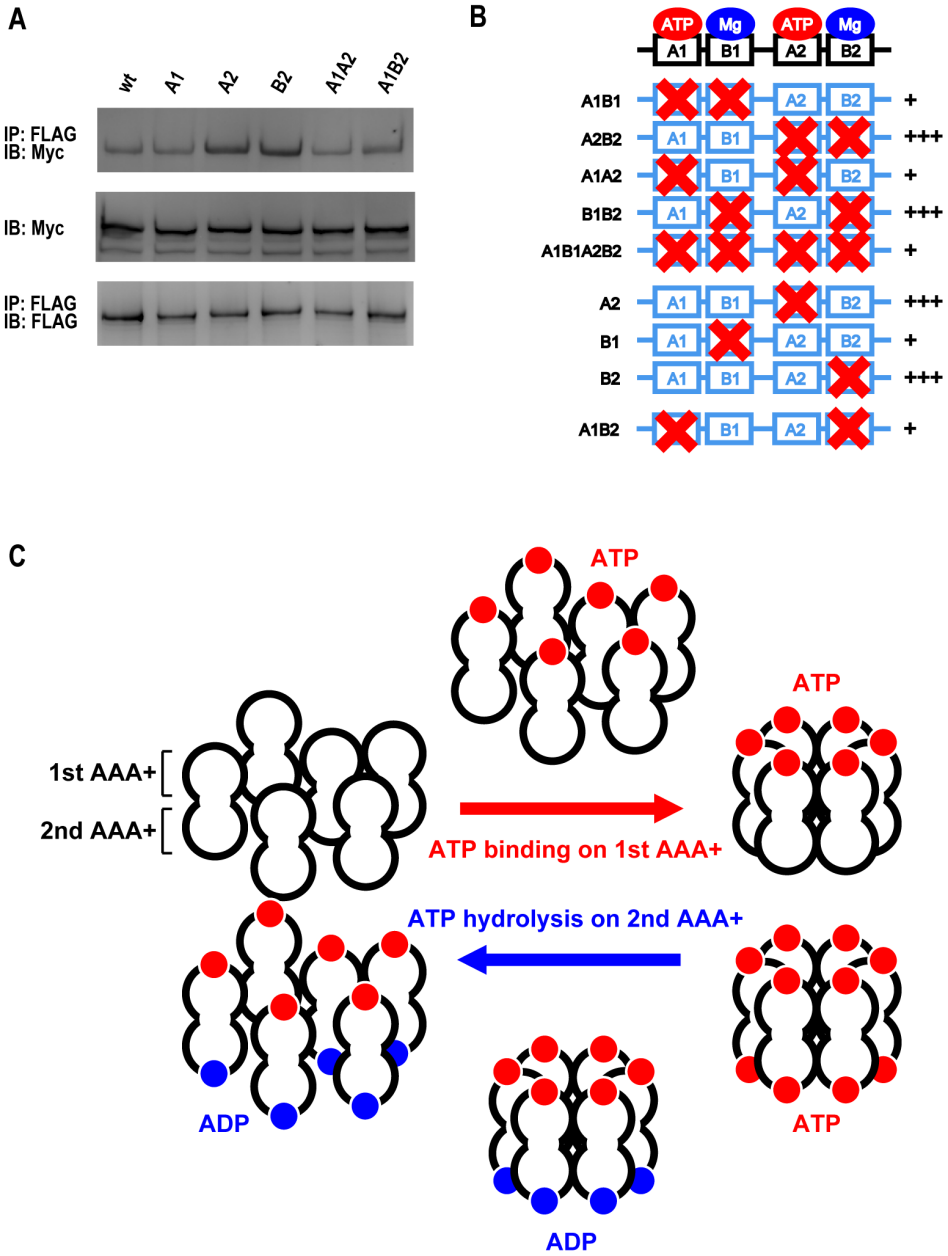
The mysterin oligomer is differently regulated by nucleotide binding to the first AAA+ module and ATP hydrolysis on the second AAA+ module. (A) A1A2 and A1B2 showed regular oligomer formation strength. Full-length blots are presented in Supplementary Fig. 4F. (B) Schematic representation of the results obtained by the oligomer formation assay. The four mutants containing mutations in the second module showed strong oligomer formation (+++), whereas A1A2, A1B2 and A1B1A2B2 showed regular assembly strength (+). (C) Model of the relationships between mysterin oligomerization and AAA+ activities.

We examined the co-IP of five combination and three single mutants and demonstrated that mutations in the second AAA+ module stabilized the oligomer through loss of ATPase activity (Fig. 4). However, some of the mutants, A1A2 and A1B1A2B2, did not exhibit such stabilization, although they also contained mutations in the second AAA+ module. This could be interpreted as an effect of mutations in the first AAA+ module; namely, the A1 and/or B1 mutation possibly canceled the effect of the mutations in the second AAA+ module. However, the B1B2 mutant still showed a stabilized co-IP (Fig. 4D), suggesting that the B1 mutation is not responsible for the potential cancellation effect. Hence, A1 was assumed to be responsible for the mutation that canceled oligomer stabilization by mutations in the second AAA+ module. To address this point, we further generated an A1B2 mutant and examined whether or not the A1 mutation canceled the B2 effect. As expected, the additional A1 mutation effectively canceled the stabilization effect of the B2 mutation (Fig. 5A). Thus, the A1, but not B1, mutation yielded a cancellation effect. As already demonstrated, the A mutation leads to the decreased ATP binding (Fig. 2B). Here, A1, but not B1, yielded a cancellation effect. Thus, nucleotide binding to the first AAA+ module is essential for oligomer stabilization. Altogether, these data suggest that mysterin oligomer formation is stabilized by ATP/ADP binding to the Walker A motif of the first AAA+ module, and is destabilized during ATP hydrolysis by the second AAA+ module (Fig. 5B and C). Such ATP/ADP binding and hydrolysis cycles could underlie potential dynamic equilibrium of mysterin.

## Discussion

In this study, we have established for the first time that mysterin belongs to the AAA+ ATPase superfamily. We previously described mysterin as a mere P-loop ATPase^14^, since the AAA+ structure surrounding the Walker motifs was not simply identified with BLAST (The Basic Local Alignment Search Tool) program. The investigation of the secondary structure of mysterin firstly allowed to identify the two AAA+ structures. It is of importance whether one protein contains simple P-loop NTPase domain or AAA+ ATPase module in considering its biological function, since the latter exhibits markedly characteristic mechanical activities. Although intracellular biological role of mysterin is still unclear, our data provides a critical basis for understanding its biological role, which is possibly mechanical and essential for proper vascular development.

It could be worth mentioning that in a previous study by another group, it was erroneously predicted that mysterin has an AAA ATPase module that consists of 114 amino acid residues (amino acids 2462–2575)^15^. As mentioned in Introduction, AAA ATPases are a subclass of AAA+ ATPases and are defined by the presence of a conserved minimum consensus sequence known as second region of homology (SRH) in addition to the specific AAA+ structure^37^,^38^. However, SRH is apparently not conserved in mysterin, and more fundamentally, a length of 114 amino acids is too short to form an AAA+ structure, which generally requires a length of approximately 200–250 amino acids. The AAA module that they predicted seems to be a mere P-loop NTPase domain.

In addition to Walker motifs, the AAA+ module contains several structural and functional elements such as the pore and sensor 1 and 2 regions^23^,^24^. The pore region is the region that composes the interior surface of the central pore of the ring-shaped AAA+ oligomer and is suggested to physically interact with substrate proteins. In cases of ClpB and Hsp104, they dissolve multipolypeptide aggregates by threading single polypeptides through their pores. The sensor 1 and 2 regions contribute to the coordination of ATPase activities of the AAA+ modules that are adjacent to each other in the ring-shaped oligomer. So far, we were not able to clearly identify these elements in mysterin AAA+ modules in this report. Definite assessment of these elements in mysterin requires more accurate structural information and in-depth biochemical studies.

The current observation suggests that mysterin possibly exists in a dynamic equilibrium between monomeric and oligomeric states, which is switched via ATP binding and hydrolysis cycles of the two AAA+ modules. This observation for the oligomer formation is consistent with the previously reported mode of assembly of AAA+ ATPases, which depends on intrinsic ATP binding and/or hydrolysis cycles, although the respective processes vary among individual AAA+ ATPases. AAA+ ATPases are postulated to exhibit specific biological activities when they form ring-shaped hexamers, whereas monomers are typically postulated to be biologically inactive. Most AAA+ ATPases form stable homo-hexamers at the physiological concentration of ATP. However, in some cases, the conversion between monomers and oligomers is regulated and is assumed to be associated with their biological roles. Katanin assembles into a homo-hexamer on its substrate microtubule filament and mediates its disassembly at the physiological concentration of ATP^34^. Once the disassembly is completed, the katanin hexamer dissociates and returns to a standby state. Vps4 is an AAA+ ATPase that essentially contributes to membrane dynamics in the endosomal pathway through disassembly and recycling of the ESCRT-III complex. Homo-hexamer formation of Vps4 is regulated by its cofactor Vta1 and also occurs on its substrate, ESCRT-III complex^39^,^40^. It is known that the homo-hexamer formation of ClpC also requires the co-factor MecA^41^. Our current data suggest that mysterin exists in a dynamic equilibrium as a huge AAA+ ATPase. Majority of mysterin protein routinely exists as an inactive monomer form, and its oligomerization is regulated by ATP/ADP binding and hydrolysis cycles. Co-factors and substrates could also contribute to the dynamic equilibrium of mysterin. Although the exact biological process in which mysterin participates has not yet been clarified, mysterin possibly plays a crucial role in vascular development through its dynamic oligomerization and mechanical activity in the cell.

## Methods

### Homology modeling

Secondary structure of the mysterin AAA+ region was predicted using network protein sequence analysis (http://npsa-pbil.ibcp.fr/cgi-bin/npsa_automat.pl?page=/NPSA/npsa_seccons.html). The homology model for the AAA+ region of mouse mysterin was obtained from RIKEN FAMSBASE, a genome-wide protein structure model database in which structural models are automatically constructed using the Full Automatic Modeling System (FAMS) based on the sequence homology with proteins whose structures have been experimentally revealed^42^. Three models of the mysterin AAA+ region that were constructed based on tandem AAA+ proteins that were found in the database (*Thermus thermophilus* ClpB, PDB code: 1QVR_C; mouse p97, 1OZ4_C; *Escherichia coli* ClpA, 1KSF_X). The structures were drawn using MOLMOL^43^.

### Plasmids

Cloning of the full-length cDNA of mysterin and generation of the disease associated variant (R4810K) were previously described^14^. The 3 × FLAG (amino acid sequence: DYKDDDDKDYKDHDGDYKDHDI, nucleotide sequence: GATTACAAGGATGACGACGATAAGGACTACAAAGACCATGACGGTGATTATAAAGATCATGACATC) and c-Myc epitopes (amino acid sequence: EQKLISEEDL, nucleotide sequence: GAACAAAAACTCATCTCAGAAGAGGATCTG) were fused to the C-terminus of mysterin using polymerase chain reaction (PCR) and were subcloned into pcDNA3.1+ (Invitrogen, California, USA) using NheI and Not I restriction sites. To generate mysterin-GFP, EGFP-C3 (Clontech, California, USA) was digested using NheI and XbaI (Thermo Fisher Scientific, Massachusetts, USA), and the clipped GFP fragment was subcloned into the N-terminus of mystein-3 × FLAG or mysterin-Myc using the NheI site (note: the NheI and XbaI sites were compatibly ligated). The first and second AAA+ fragments (a.a. 2359–2613 and 2718–2972, respectively) were subcloned into pGEX5X-1 (Addgene, USA) using SmaI and XhoI sites (note: the first AAA+-GST was described in our previous paper^14^). Four mutations [A1: K(AAA)2426A(GCA), B1: E(GAA)2488A(GCA), A2: K(AAG)2775A(GCG), and B2: E(GAG)2845A(GCG)] were introduced into mysterin-3 × FLAG, mysterin-Myc, AAA+ fragments fused with GST using Pfu Turbo DNA polymerase (Agilent Technologies, California, USA) and restriction enzyme DpnI (New England BioLabs, Massachusetts, USA), according to the manufacturer's instructions for the QuickChange site-directed mutagenesis kit (Agilent Technologies, USA).

### Cell culture and transfection

HEK-293 cells and Neuro2A cells were maintained in DMEM (Life Technologies, California, USA and Sigma Aldrich, Missouri, USA, respectively) supplemented with 10% fetal bovine serum, 100 U/mL penicillin, and 100 μg/mL streptomycin. Each 0.5 μg plasmid was transfected into HEK293 or Neuro2A cells using Lipofectamine LTX (Life Technologies) or 2000 (Life Technologies), according to the manufacturer's instruction.

### Protein preparation and ATPase assay

The first and second AAA+ ATPase modules that are fused with GST were purified using GSH beads (GE Healthcare, USA) as previously described^14^. ATPase activity was measured by malachite green assay using BIOMOL Green kit (Enzo Life Sciences, USA) as previously described^14^,^44^.

### Protein preparation and electron microscopy

Mysterin-3 × FLAG was transiently transfected into HEK293 cells. Cells were harvested 24 h after the transfection and were treated with lysis buffer containing 1% NP-40, 150 mM KCl, and 50 mM HEPES-NaOH (pH 7.5). The lysate was incubated with anti-FLAG M2 affinity gel (Sigma Aldrich). Captured mysterin-3 × FLAG was eluted using lysis buffer containing 0.4 mg/mL 3 × FLAG epitope (Sigma Aldrich) following repeated washing with the lysis buffer. A total of 100 μL affinity beads and 250 μL elution buffer were used for approximately 10^8^ cells. Electron micrographs were recorded on a JEOL 1010 equipped with a 2 K slow-scan charge-coupled device (CCD) camera (Gatan, California, USA) with a magnification of 40 K and operated at 100 kV. The specimens were negatively stained with 2% uranyl acetate.

### Antibodies, IP, and ATP-binding assay

Anti-FLAG (mouse monoclonal M2; Sigma Aldrich), anti-Myc (mouse monoclonal 9E10; Santa Cruz Biotechnology, Texas, USA) and anti-GST (goat polyclonal; GE Healthcare) antibodies were purchased. For IP of mysterin-3 × FLAG, the cell lysate was incubated with anti-FLAG M2 affinity gel (Sigma Aldrich). For IP of mysterin-Myc, the cell lysate was incubated with anti-Myc antibody and subsequently with protein G beads (GE Healthcare AB, Uppsala, Sweden). For ATP-binding assay, purified protein was incubated with ATP agarose (high) (Innova Biosciences, UK). The beads were repeatedly washed with lysis buffer, as described above. Subsequently, they were eluted in Laemmli's sample buffer and were analyzed using SDS-PAGE following Western blotting using the above antibodies as previously described^45^.

### Fluorescence correlation spectroscopy

FCS measurements were performed using a ConfoCor 2 system combined with LSM 510 META (Carl Zeiss, Jena, Germany) through a C-Apochromat 40×/1.2NA UV-VIS-IR Korr water-immersion objective (Carl Zeiss). A confocal pinhole diameter was adjusted to 70 μm. EGFP was excited at 488 nm and emission signals were detected using a 505-nm long-pass filter. Measurements were performed in a glass-based cell culture dish (Asahi glass Co., Ltd., Tokyo, Japan) that was incubated at 37°C. The fluorescence autocorrelation function, 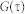, from which the average correlation time (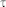) and the absolute number of fluorescent proteins in the detection volume were calculated, were obtained using the Equation 1 as follows: 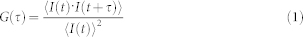
where 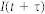 is the fluorescence intensity obtained by single-photon counting method in a detection volume at a delay time (brackets denote ensemble averages).

Curve fitting for the two-component model was given by Equation 2 as follows: 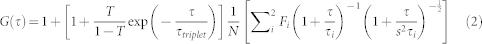
where *F_i_* and 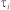 are the fraction and diffusion time of component *i*, respectively; *N* is the average number of fluorescent molecules in the detection volume defined by the beam waist *w*_0_ and the axial radius *z*_0_; *s* is the structure parameter representing the ratio of *w*_0_ and *z*_0_; *T* is the triplet fraction; and 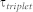 is the relaxation time of the triplet state. 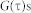 of living cells were measured for 60 s. Following pinhole adjustment, the diffusion time (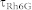) and structure parameter (*s*) were determined using a 10^−7^ M Rhodamine6G (Rh6G) solution as a standard before measurements. The diffusion coefficient was determined using Equation 3 as follows: 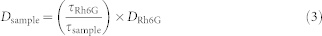
where *D*_sample_ and *D*_Rh6G_ ( = 414 μm^2^/s) are the diffusion coefficient of the sample and Rh6G, respectively. The molecular weight of Mst-GFP was calculated using Equation 4, which was derived from the Stokes–Einstein relation, as follows: 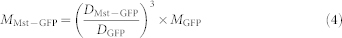
where *M*_Mst-GFP_ and *M*_GFP_ ( = 27 kDa) are the molecular weight of Mst-GFP and monomeric GFP, respectively. Counts per molecule (CPM) value is calculated as a ratio of 〈*I*(t)〉 to N.

## Author Contributions

D.M. and K.N. designed the study and wrote the manuscript. D.M., K.N., A.K., Y.K. and K.K. performed experiments. D.M., J.H., M.K., Y.F. and K.N. analyzed the data. All authors reviewed the manuscript.

## Supplementary Material

Supplementary Information supplementary information

## Figures and Tables

**Table 1:** Curve-fitting results of FCS measurements in living cells

	CPM (kHz)	Fast diffusion coefficient (μm^2^/s)	Fast fraction (%)	Slow diffusion coefficient (μm^2^/s)	Slow fraction (%)	Measured cell number
mysterin-GFP	* 3.2 ± 1.3	* 13.4 ± 3.0	* 43.6 ± 5.5	0.49 ± 0.25	* 56.4 ± 5.5	17
GFP	* 5.6 ± 0.84	* 36.4 ± 5.9	* 94.9 ± 1.5	0.87 ± 0.71	* 5.1 ± 1.5	7

*p < 0.01 by Student's t-test.
