# Supplementary material for: Moyamoya disease-associated protein mysterin/RNF213 is a novel AAA+ ATPase, which dynamically changes its oligomeric state
Source: Sci Rep. 2014 Mar 24;4:4442. doi: 10.1038/srep04442 (PMC3963067; doi:10.1038/srep04442)
Supplement: Supplementary Information [file srep04442-s1.pdf]

Supplementary Information

Moyamoya disease-associated protein mysterin/RNF213 is a novel AAA+ ATPase, which dynamically changes its oligomeric state

Daisuke Morito[a], Kouki Nishikawa[b], Jun Hoseki[c,d], Akira Kitamura[e], Yuri Kotani[a], Kazumi Kiso[a], Masataka Kinjo[e], Yoshinori Fujiyoshi[b] and Kazuhiro Nagata[a,1]

[a]Laboratory of Molecular and Cellular Biology, Faculty of Life Sciences, Kyoto Sangyo University, Kyoto 603-8555, Japan
[b]Cellular and Structural Physiology Institute, Nagoya University, Nagoya, 464-8601, Japan
[c]Research Unit for Physiological Chemistry, The Center for the Promotion of Interdisciplinary Education and Research, Kyoto University, Kyoto, 606-8502, Japan
[d]Division of Applied Life Sciences, Graduate School of Agriculture, Kyoto University, Kyoto, 606-8502, Japan
[e]Laboratory of Molecular Cell Dynamics, Faculty of Advanced Life Science, Hokkaido University, Sapporo, 001-0021, Japan

[1]Correspondence:
Kazuhiro Nagata
Laboratory of Molecular and Cellular Biology,
Faculty of Life Sciences,
Kyoto Sangyo University
Kamigamo Motoyama
Kyoto 603-8555, Japan
+81-75-705-3121
nagata@cc.kyoto-su.ac.jp

Supplementary Fig. 1. Organization of the secondary structures of mysterin resembles those of AAA+ proteins. (A) Secondary structure of the mysterin 1st AAA+ module is compared with those of ClpB and p97. (B) Secondary structure of the mysterin 2nd AAA+ module is compared with those of ClpB and p97. The α helices and β sheets are indicated with h and e, respectively; c indicates a nonstructural region.

Supplementary Fig. 2. FCS measurement of GFP-tagged mysterin in living Neuro2A cells. (A) A typical image of Mst-GFP observed on confocal fluorescence microscopy. The white dot emphasized by the arrow shows the position of FCS measurement in the cell. Scale = 5 μm. (B) Typical recording of the fluorescence fluctuation of Mst-GFP obtained by live-cell FCS measurement. (C) Typical recording of the fluorescence fluctuation of GFP obtained by live-cell FCS measurement. (D) Normalized autocorrelation function, $G(\tau)$, of Mst-GFP (green) and GFP (red).

Supplementary Fig. 3. Oligomer formation of the disease-associated variant (R4810K). The complex formation of wild type (wt) or R4810K variant mysterin was examined as schematically represented in Fig. 4A.

Supplementary Fig. 4. Original full-length blots before cropping. (A) Original blot for Fig. 2B. (B) Original blots for Fig. 4B. Immunoprecipitated samples (left upper) and lysate (left lower) were transferred onto the same membrane (left) to estimate the efficiency of immunoprecipitation as described in the main text. (C) Original blots for Fig. 4D. (D) Original blots for Fig. 4E. (E) Original blots for Fig. 4F. (F) Original blots for Fig. 5A.

Supplementary Figure. 1A

Morito et al.

Mysterin 1st AAA+ module

```
                                                                                          Walker A: GETGCGKT
Mysterin    RHKKLERLCLTLGIPQATDPDKTYELTTDNMLKILAIEMRFRCGIPVIIMGETGCGKTRL
DSC         hhhhhhhhhheeccccccccccccchhhhhhhhhhhhheecccccceeeecccccccchh
MLRC        hhhhhhhhhhhhccccccccccceeechhhhhhhhhhhhhhcccceeeeeeccccchhhh
PHD         hhhhhhhhhhhccceccccccccceeecchhhhh[α0]hhhhhcccceee[β1]ecccccchhhhh
Predator    hhhhhhhhhhhhcccccccccceeeeccchhhhhhhhhhhhhcccceeeeeccccccccc
Sec.Cons.   hhhhhhhhhhhhccccccccccceeec?hhhhhhhhhhhhhcccc?eeee?ccccc??hh

ClpB        cchhhhhheeehhhhhhhcccccccchhhhhhhhhhhhhhcccceeeee-ccccchhhhh
p97         cccccccchhhhhh--hhhhhccccccccccc-----cccccccceeeecccccccchhhh

                                                                                          Walker B: ILFFDE
Mysterin    IKFLSDLRRGGTNADTIKLVKVHGGTTADMIYSRVREAENVAFANKDQHQLDTILFFDEA
DSC         hhhhhhhcccccccchheeeeecccccchhhhhhhhhhhhhhhhhccccccceeeehhhc
MLRC        hhhhhhhhccccccceeeeeeecccccchhhhhhhhhhhhhhhhcccccccceeeeeeccc
PHD         hhh[α1]hhhhccccceee[β2]eecccccchhhhhhh[α2]hhhhhhhccccccceee[β3]eecc
Predator    chhhhhcccccccccceeeeeeeccccchhhhhhhhhhhhhhhhhccccccceeeeeccc
Sec.Cons.   hhhhhhh?ccccccceeeeeeeccccc?hhhhhhhhhhhhhhhh?cccccc?eeeeeccc

ClpB        hhhhhhhhhhhccccccccceeeeeccxxxxxxhhhhhhhhhhhhhhhcccceeeeeccx
p97         hhhhhhhhcc-----eeeeeeccccc--ccccccchhhhhhhhhhhhhhccceeeeeecc

Mysterin    NTTEAISCIKEVLCDHMVDGQPLAEDSGLHIIAACNPYRKHSEEMICRLESAGLGYRVSM
DSC         chhhhcchhhhhhhhcccccccccccccccccccccccccchhhhhhhhhhhcccccccc
MLRC        cchhhhhhhhhhhhcccccccccccccceeeeecccccccchhhhhhhhhhcccceeeeh
PHD         cchhhh[α3]hhhhhcccccccccccccceee[β4]ecccccccchhh[α4]hhhhcccce[β5]c
Predator    cccceeeeeeeeeccccccccccccccccceeeeeccccchhhhhhhhhhhccccccccch
Sec.Cons.   cchhhhhhhhhhhhcccccccccccccc?eeee?ccccccchhhhhhhhh?cccc???c?

ClpB        xxxxxxxxxxxxxxxxxxchhhhh-hcccceeeeehhhhhhh-hhchhhhhhce-eeeee
p97         ccccccccchhhhhhhhhhhcccccccccceeeeccccc----ccccccccccccceeec

Mysterin    EETADRLGSIPLRQLVYRVHALPPSLIPLVWDFGQLSDVAEKLYIQQIVQRLVESISLDE
DSC         cccccccccchhhhhhhhhcccccccchhhhhhccccchhhhhhhhhhhhhccccccccc
MLRC        hhhhhhhcccchhhhhhhhccccchhhhhhhcccccchhhhhhhhhhhhhhhhhhcccch
PHD         ccccccccccccee[α5]eeeccchhhhh[α6]hhhhccccchhhhhh[α7]hhhhhhhccccccc
Predator    hhhhhhccchhhhhhhhhhhhcccccceeeeeccccchhhhhhhhhhhhhhhhhhhhcccc
Sec.Cons.   ??????cccc?hhhhhhhhccccc??hhhhh??cccc?hhhhhhhhhhhhhhh??ccccc

ClpB        ccccccchhhhhhhhhhhhhhhhcceehhhhhhhhhhhhhhhhccchhhhhhhhhhhhcc
p97         ccccchhhhhhhhhhhcc--ccccccccchhhhhccccccccchhhhhhhhhhhhhhhhh

Mysterin    NGTRVITEVLCASQGFMRKTEDE
DSC         ccchhhhhhhhhhhhhhhhcccc
MLRC        chhhhhhhhhhhhhhhhhccccc
PHD         chhhhhhhh[α8]hhhhhhhhhccc
Predator    ccceeeeeecccccccccccccc
Sec.Cons.   c??hhhhhhhhhhhhhhh?cccc

ClpB        chhhhhhhhhhhcchhhhhhhhh
p97         hhhhcccc--cccchhhhhhccc
```

Supplementary Fig. 1B

Morito et al.

Mysterin 2nd AAA+ domain

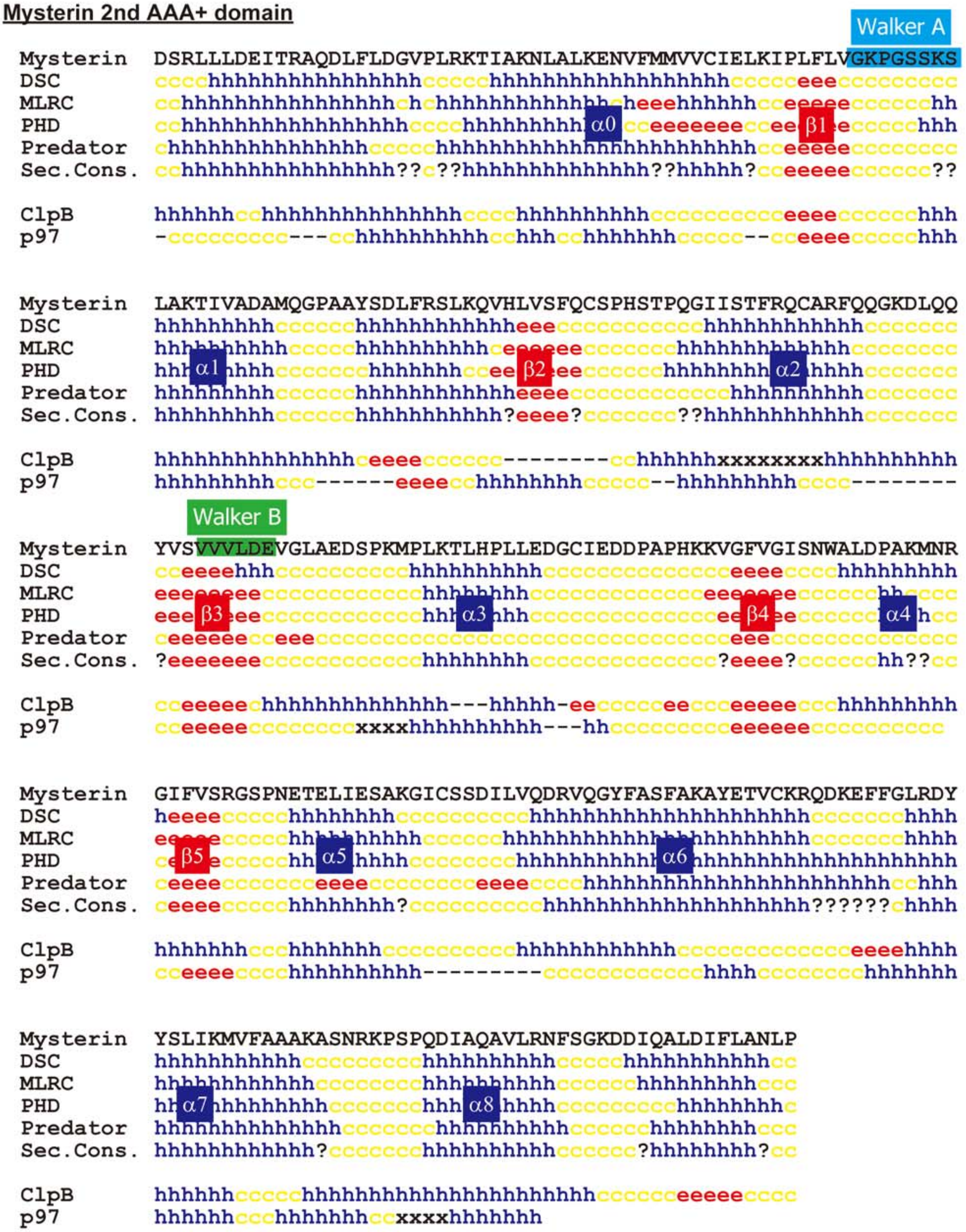

Supplementary Figure 2

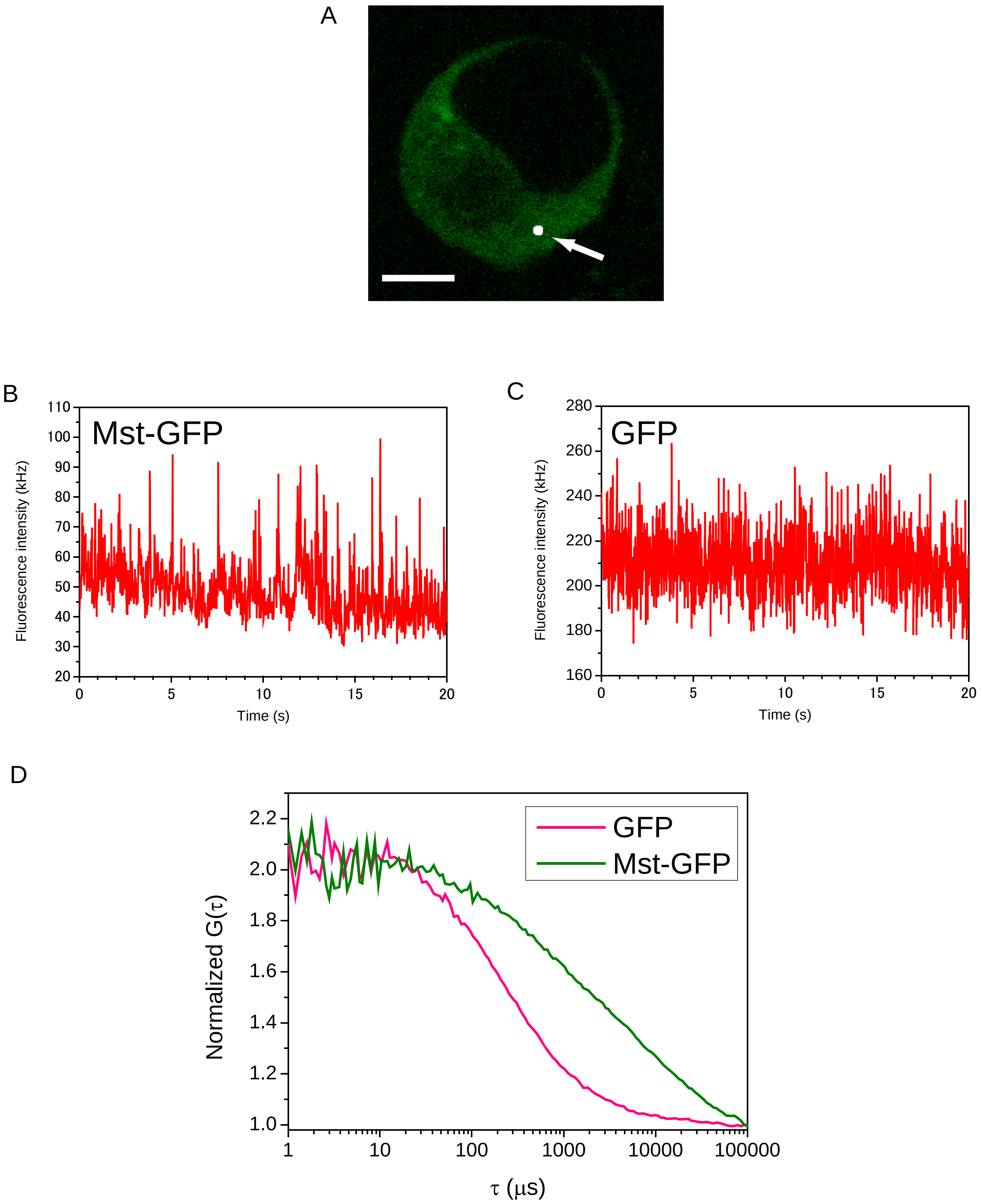

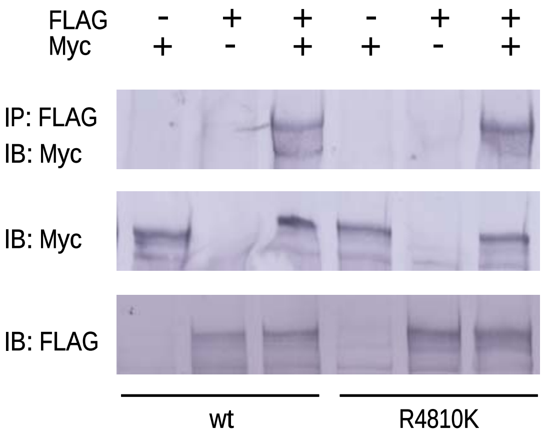
FLAG - + + - + +
Myc + - + + - +
IP: FLAG
IB: Myc
IB: Myc
IB: FLAG
wt
R4810K

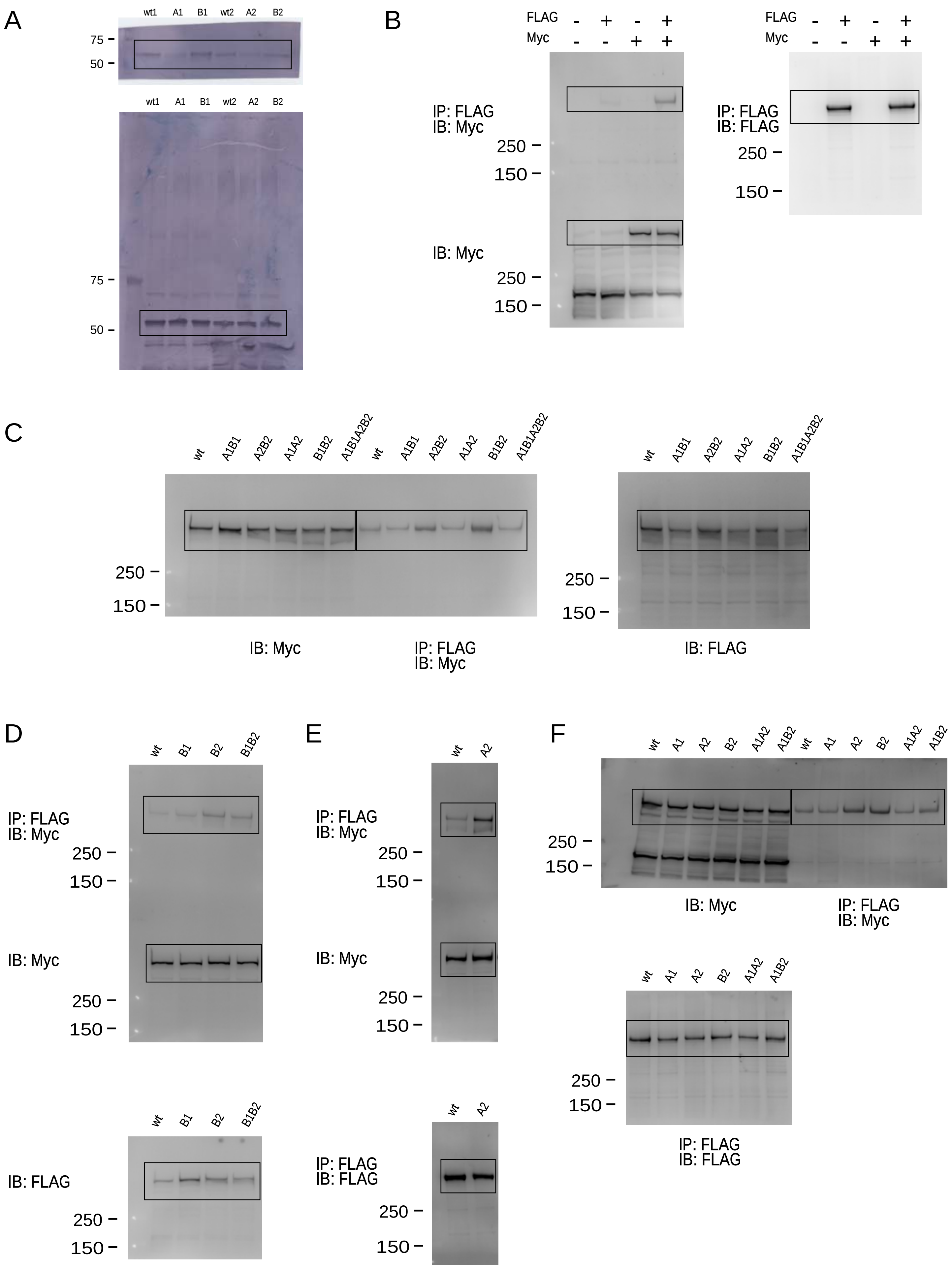
A
wt1 A1 B1 wt2 A2 B2
75
50
wt1 A1 B1 wt2 A2 B2
75
50
B
FLAG - + - +
Myc - - + +
IP: FLAG
IB: Myc
250
150
IB: Myc
250
150
FLAG - + - +
Myc - - + +
IP: FLAG
IB: FLAG
250
150
C
wt A1B1 A2B2 A1A2 B1B2 A1B1A2B2 wt A1B1 A2B2 A1A2 B1B2 A1B1A2B2
250
150
IB: Myc
IP: FLAG
IB: Myc
wt A1B1 A2B2 A1A2 B1B2 A1B1A2B2
250
150
IB: FLAG
D
wt B1 B2 B1B2
IP: FLAG
IB: Myc
250
150
IB: Myc
250
150
wt B1 B2 B1B2
IB: FLAG
250
150
E
wt A2
IP: FLAG
IB: Myc
250
150
IB: Myc
250
150
wt A2
IP: FLAG
IB: FLAG
250
150
F
wt A1 A2 B2 A1A2 A1B2 wt A1 A2 B2 A1A2 A1B2
250
150
IB: Myc
IP: FLAG
IB: Myc
wt A1 A2 B2 A1A2 A1B2
250
150
IP: FLAG
IB: FLAG
